# News, New products, Announcement

**DOI:** 10.1155/S1463924601000268

**Published:** 2001


Journal of Automated Methods & Management in Chemistry, Vol. 23, No. 6 (November–December 2001) pp. 193–194

### News

### MDS Pharma Services enhances scientific capabilities with API 4000 LC/MS/MS system

*Montreal, Quebec—16 August 2001*: MDS Pharma Services has become the first contract research organization to purchase the API 4000 LC/MS/MS System from Applied Biosystems/MDS Sciex. The API 4000 is at the pinnacle of triple quadrupole platforms, which are used in bioanalytical research during the drug discovery and development process.

The API 4000 system creates a new standard for instrumentation ruggedness and reliability, enabling even greater productivity in the laboratory. It presents a tenfold increase in sensitivity for many analytes, compared with other triple quadrupole LC/MS/MS systems.

'We have, in fact, plans in place for several API 4000 LC/MS/MS systems,' said Jim Hulse, senior vice president, bioanalytical, MDS Pharma Services. 'The capabilities of the API 4000 are of critical importance. More and more drugs with highly specific pharmacological activities and lower daily dosage recommendations are being developed through pharmaceutical pipelines. The lower the dosage recommendation, the greater the sensitivity needed in laboratory instrumentation to analyse samples. The API 4000 will be crucial in determining the safety and efficacy of many new drugs.'

The system is fully integrated with powerful data acquisition and processing capabilities. As a result, the system provides much faster access to information than its predecessors. It represents the best detection limit and highest throughput capacity available. It also enables automated MS to MS/MS acquisition of data for maximum extraction of information from a single LC/MS run.

'Our first API 4000 is now being employed in our laboratory in St. Laurent, Quebec,' continued Hulse. 'We are encouraging our clients to send us their most difficult bioanalytical research assignments, as the API 4000 will most certainly be able to help.'

### MDS Pharma Services announces addition of BIACORE 3000 System

*Montreal, Quebec—16 October 2001*: MDS Pharma Services announced today that it has purchased the BIACORE 3000 instrumentation system for use in bioanalytical research, including drug discovery assignments. The new BIACORE 3000 instrumentation system represents a significant advancement in the automation of bioanalytical research. It also provides highly advanced sensitivity in sample analysis and vital flexibility in method development.

'The BIACORE 3000 is the highest performance system available for label free studies of biomolecular binding,' said Jim Hulse, senior vice president, bioanalytical, MDS Pharma Services. 'The system represents a significant investment. This is another example of our dedication to enhancing the capabilities in our bioanalytical laboratories. A key driver to our business is the dedication to staying on the forefront of technology. This specific investment in the BIACORE 3000 will help us answer vital questions about the speed, strength, and specificity of biomolecular binding and determine the active concentrations of components. The speedy and accurate acquisition of such data is crucial to our clients.'

MDS Pharma Services is employing the new BIACORE 3000 in its bioanalytical laboratory in St. Laurent, Quebec, Canada.

Various technical innovations incorporated into the BIACORE 3000 allow it to meet the highest demands for sensitivity, efficiency, and flexibility. The system shortens analysis times, minimizes sample consumption, works with both non-aqueous and aqueous samples, efficiently develops non-standard applications, and performs the most advanced kinetic evaluations.

'One of the key strengths of the BIACORE 3000 is that it allows us to quickly develop non-standard applications,' continued Hulse 'It gives us complete freedom to develop fully customized methods that are then quickly ready-to-run.'

The BIACORE 3000 was developed and manufactured by Biacore International AB, globally headquartered in Sweden. Biacore is a global leader in the development, manufacture and marketing of innovative and unique products to detect and monitor molecular binding. Biacore systems are used in drug discovery, life science research, and food analysis.

As the global leader in bioanalytical services, MDS Pharma Services offers extensive expertise in method development, rapid sample analysis, custom assay development, biochemical markers, and statistical and pharmacokinetic services. This advanced technology, along with highly-skilled scientific staff and extensive emphasis on customer service, ensure reliable client solutions. With bioanalytical laboratories worldwide, equipped with more than 70 LC/MS/MS instruments, and over 700 available assays, MDS Pharma Services rapidly delivers accurate results.

MDS Pharma Services offers a full spectrum of resources to meet the drug discovery and development needs of the pharmaceutical and biotechnology industries. With facilities in more than 18 countries, the company applies advanced scientific and technological expertise to each stage of the drug discovery and development process: discovery, preclinical, early clinical research (phases I-IIa), bioanalytical, global clinical research (phases IIb-IV), and central lab.

MDS Pharma Services is part of MDS Inc. (TSE: MDS; NYSE: MDZ), an international health and life sciences company. In many of its products and services, it is among the largest and most respected companies in the world. At MDS, the focus is on fighting disease. It does this by providing laboratory testing, imaging products, and research services to speed discovery and development of new drugs, therapy systems for planning and delivery of cancer treatments, analytical instruments to assist in the development of new drugs, and medical/surgical supplies. MDS employs more than 10 000 highly skilled people in its global operations on five continents. Detailed information about the company is available at the MDS Web site at www.mdsintl.com or by calling +1 (888)-MDS-7222, 24 hours a day.

*For further information please contact Phil Tegeler, MDS Pharma Services, 621 Rose Street, Lincoln, NE, USA. Tel: +1 (402)-476-2811, and visit our Web site at www.mdsps.com*

Journal of Automated Methods & Management in Chemistry, Vol. 23, No. 6 (November–December 2001) pp. 195–196

### New products

### New manual rotary distribution valves—high precision fluid and gas control

New from Omnifit is a range of large manual rotary distribution valves for high precision gas and fluid control in laboratory equipment and analytical instruments.

Pressure rated up to 500 psi (33 bar), the new valves have chemically inert PTFE wetted parts, sturdy PEEK casings and KEL-F rotors. There are 5-, 7-, 9- and 11-port versions for 4-way, 6-way, 8-way and 10-way distribution, as well as 6- and 10-port versions for loop injection. Port connections are standard 1/4–28 UNF threads and port sizes are standard 1.5 mm. Each port is identified by a sample click-stop.

*For further information please contact: Robin Higgons, Omnifit Ltd, 2 College Park, Coldhams Lane, Cambridge CB1 3HD, UK. Tel: +44 (0)1223 416642; Fax: +44 (0)1223 416787; e-mail: sales@omnifit.co.uk; Website: www.omnifit.co.uk*

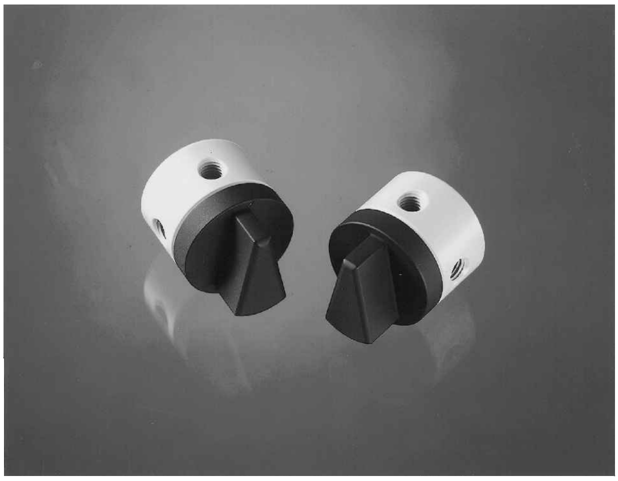

*New manual rotary distribution valves.*

### The financial benefits of process monitoring with NIR

The financial benefits of using near infrared spectroscopy (NIR) as a process monitoring tool in many industries, including pharmaceutical, chemical and food, have led many larger companies to invest and reap the benefits of fast response times, increased throughput, less re-work, improved quality, etc.

Process Instruments (UK) Ltd are worried that smaller companies are put off using NIR owing to the perceived difficulties of developing calibrations and maintaining the instruments.

Advances in NIR technology made by LT Industries Inc. (whose products are supplied in the UK by Process Instruments), mean that calibration development on the latest NIR spectrometers is relatively simple and doesn't require the support of a large laboratory that may only be available to larger organizations.

Installation and servicing of the instruments is relatively simple, with the instruments capable of being used on-line, in-line and at-line, even in hazardous areas. Sophisticated process control outputs are available and data can even be made available over the Internet.

In order to dispel the myths surrounding the development of calibration models, Process Instruments are offering to develop rudimentary models, free of charge, to any company that would like to see whether or not NIR will allow them to improve their process.

Some of the processes that can be monitored are OH number, distillation, raw material ID, textile fibres, organic addition, acid number, protein content, fat content, moisture context, and thin films.

*For further information please contact: Mike Riding, Process Instruments (UK) Ltd, Process House, Dominion Court, Billington Road, Burnley, Lancashire BB11 5UB, UK. Tel: +44 (0)1282 422835; Fax: +44 (0)1282 422268; e-mail: processinsts@cs.com*

### New Carousel PTFE Gas-Tight Threaded Cap from Radleys Discovery Technologies performs better—even with aggressive solvents

Radleys Discovery Technologies (RDT) has introduced a brand new PTFE cap for use with its popular Carousel Reaction Station.

The new cap, which is produced with a PTFE valve and 'barbed' stainless steel inlet, offers significant benefits over the previous model—at the same price!

- Suitable for use with the most aggressive solvents.
- No risk of corrosion.
- Easier to operate.
- Barbed side arm designed for easy removal of tubing.

The cap is suitable for use with the Carousel Reaction Station, Cooled Reaction Station and new Metz Syn10 Variable Reaction Station.

### Truly versatile parallel chemistry

Ideal for chemists involved in all aspects of reaction optimization and process development, the new Metz $Syn^{10}$ from Radleys Discovery Technologies will simultaneously carry out 10 reactions, each with a different temperature, from $-30$ to $+150\,^{\circ}C$.

The Metz $Syn^{10}$ is the latest addition to the popular Metz range of benchtop reaction stations, offering truly versatile parallel synthesis with variable heating, cooling, refluxing, inert gas and stirring.

Developed in collaboration with leading pharmaceutical companies, the Metz Syn$^{10}$ is ideal for a wide range of applications including reaction and process optimization, parallel synthesis, combinatorial chemistry, chemical and catalyst development.

The Syn$^{10}$ is remotely controlled via the RS232 interface by either a dedicated palm-top or personal computer. Each of the 10 wells may be programmed with an individual reaction profile. You can program any number of temperatures or stirring ramps or dwells for any reaction duration, then store each reaction profile for use as required.

The Syn$^{10}$ offers stirring control from 25 to 1200 rpm, with a maximum temperature difference between any two adjacent wells of an amazing 180 °C.

An optional removable water-cooled Reflux unit, based upon the successful 'Carousel refluxing system' is used to provide even and efficient refluxing within the glass reaction tubes, minimizing solvent evaporation during synthesis for even the most volatile solvents—such as ether.

Unique PTFE caps with PTFE valves combine with an integral gas distribution system to allow the introduction of inert gas into the reaction for air or moisture sensitive reactions.

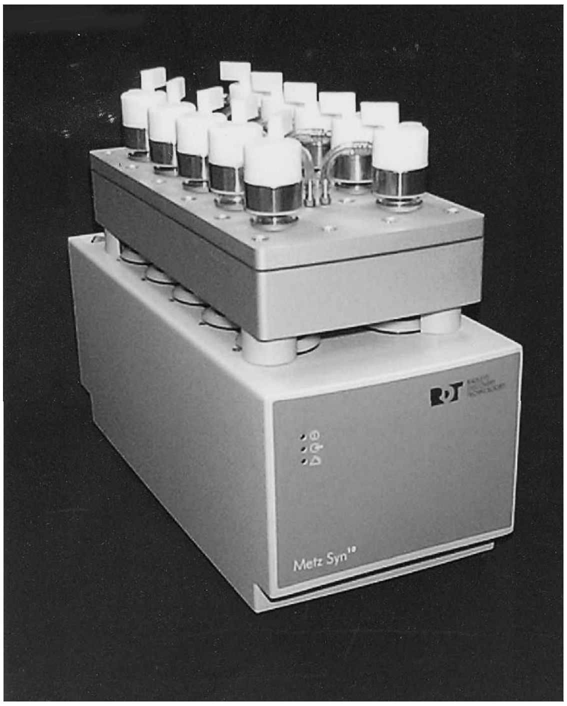

*Other features*

- Stirring ramp/soft start to ensure stirrer bar coupling.
- Compatible with Carousel tubes, caps and stirring bars.
- Clearly numbered reaction positions.
- Use of all or some positions.

With the ability to vary each reaction by temperature, solvent, order of reagent, etc., your chemistry can be fine-tuned to optimize the reaction steps and allow evaluation to maximize yields or develop different routes.

The new Metz Syn$^{10}$ is available worldwide through RDT's network of specialist distributors.

*For more information or to arrange a demonstration, please contact Clare Schneider, Marketing Co-ordinator, Radleys Discovery Technologies, Shire Hill, Saffron Walden, Essex CB11 3AZ, UK. Tel: +44 (0)1799 513320; Fax: +44 (0)1799 513283, e-mail: clare.schneider@radleys.co.uk; Web: www.radleys.com*

### Announcement

### Funding helps SME's accelerate in lean manufacturing

Funding to help small and medium sized manufacturing companies leap forward into lean manufacturing is available through The Manufacturing Institute's Accelerated Route to Lean Manufacturing. Expressly created for manufacturers who are seeking new ways to boost shop floor performance, improve productivity and reduce costs, the programme takes manufacturing professionals through a series of highly practical, interactive and applied lean manufacturing workshops. Its aim is to give manufacturing professionals their very own lean toolkit enabling them to implement lean manufacturing back at base.

Thanks to a grant from the European Social Fund (ESF), small and medium sized manufacturers in the North West of England will receive a heavy subsidy covering 80% of the costs for the programme.

'The Accelerated Route to Lean Manufacturing is an active and practical response to driving out organizational waste in today's turbulent manufacturing environment where companies face a continuous need to reduce costs, add value for customers and deliver total flexibility,' says Julie Madigan, Chief Executive of The Manufacturing Institute. 'We are absolutely delighted that the European Social Fund is subsidising this much needed programme for SME manufacturing companies in the North West.'

During the programme, involving one day a week's 'learning by doing', delegates are coached by some of Britain's most highly acclaimed manufacturing experts and industry practitioners in a whole spectrum of tried and tested techniques. The accent is placed on waste elimination, defect prevention, customer pulled production, teamworking, motivation, solving root cause problems, quality in the eyes of the customer, supply chain development and performance measurement. To promote sustainability of lean activities in the company, those that go through the Accelerated Route to Lean Manufacturing can opt to undertake some in-company improvement work that focuses on particularly high priority problem areas. This provides an opportunity for greater application of lean thinking to the company for business benefit and for individuals to gain recognition for their achievements through the award of The Manufacturing Institute's Fellowship in Lean Manufacturing.

Following completion of the programme, up to ten days of on-site practitioner help drawn from The Institute's extensive network of high-achieving lean experts is also available at a subsidized rate for those who need further assistance back at base.

*For more information contact Jo Britton at The Manufacturing Institute on +44 (0)161 874 3204, e-mail: joannab@tpmi.co.uk or visit www.manu-online.com*

